# Dreaming of motherhood: experiences of women who have undergone fertility treatment

**DOI:** 10.17533/udea.iee.v43n1e06

**Published:** 2025-04-28

**Authors:** Sara Rujas Bracamonte, Pilar Serrano Gallardo, Mercedes Martínez Marcos

**Affiliations:** 1 Midwife, Ph.D. Hospital Universitario de Móstoles. Móstoles, Madrid, Spain. Email: sara.rujas@salud.madrid.org. https://orcid.org/0000-0003-0140-4499 Hospital Universitario de Móstoles Madrid Spain sara.rujas@salud.madrid.org; 3 Nurse, Ph.D. Email: mercedes.martinezmarcos@uam.es. https://orcid.org/0000-0002-5163-6821 Universidad Autónoma de Madrid Spain mercedes.martinezmarcos@uam.es; 4 Nursing Department, Faculty of Medicine, Universidad Autónoma de Madrid (UAM). Health Research Institute Puerta de Hierro Majadahonda (IDIPHISA), Madrid, Spain. https://orcid.org/0000-0001-8603-7813 Universidad Autónoma de Madrid Nursing Department Faculty of Medicine Universidad Autónoma de Madrid (UAM) Madrid Spain

**Keywords:** infertility, reproductive techniques, assisted, grounded theory, research, qualitative, women., infertilidad, técnicas reproductivas asistidas, teoría fundamentada, investigación cualitativa, mujeres**.**, infertilidade, técnicas de reprodução assistida, teoría fundamentada, pesquisa qualitativa, mulheres.

## Abstract

**Objective.:**

to describe the strategies used by women who have undergone assisted reproductive technologies (ART) to cope with the process of becoming mothers and to describe their relationships with their partners and healthcare professionals throughout this process.

**Methods.:**

This is a qualitative study based on grounded theory. Twenty women who had undergone ART in Spain were selected. Semi-structured interviews were used to collect the data. The analysis followed grounded theory methods.

**Results.:**

‘Fighting for a dream: motherhood’ is the main category describing women’s struggles from their diagnosis of infertility to their successful pregnancy and delivery. This process makes it possible for them to fulfil their dream and become parents along with their partners. Three subcategories describe the different stages in the process: ‘Accepting treatment: doing everything possible’, ‘Undergoing treatment: an emotional rollercoaster’, and ‘Reconsidering the dream: give up or gather strength to keep going?’

**Conclusion.:**

Women undergoing ART experience difficulties as they seek to achieve pregnancy, as there is no guarantee that they will be able to fulfil their greatest desire in life: motherhood. Women use a variety of coping strategies during ART and continue to seek emotional support from their peers and/or women who are experiencing the same situation.

## Resumen

**Objetivo.:**

Describir las estrategias utilizadas por las mujeres que se han sometido a un tratamiento de fertilidad para afrontar el proceso de ser madres, y describir sus relaciones con sus parejas y profesionales sanitarios a lo largo de este proceso.

**Métodos.:**

Se trata de un estudio cualitativo basado en la teoría fundamentada. Se seleccionaron 20 mujeres que se habían sometido a Tecnología de Reproducción Asistida (TRA) en España. Se utilizaron entrevistas semiestructuradas para recoger los datos. El análisis siguió los métodos de la teoría fundamentada.

**Resultados.:**

“Luchar por un sueño: la maternidad” es la categoría principal que describe la lucha de las mujeres desde su diagnóstico de infertilidad hasta su embarazo y parto exitosos. Este proceso les permite cumplir su sueño y convertirse en padres junto a sus parejas. Tres subcategorías describen las distintas etapas del proceso: “Aceptar el tratamiento: hacer todo lo posible”, “Someterse al tratamiento: una montaña rusa emocional” y “Reconsiderar el sueño: ¿renunciar o reunir fuerzas para seguir adelante?”

**Conclusión.:**

Las mujeres que se someten a TRA experimentan dificultades al intentar conseguir un embarazo, ya que no hay garantías de que puedan cumplir su mayor deseo en la vida: la maternidad. Las mujeres utilizan diversas estrategias para mejorar el afrontamiento durante los TRA y continúan buscando apoyo emocional por parte de su entorno y/o de mujeres que están experimentando la misma situación.

## Resumo

**Objetivo.:**

Descrever as estratégias utilizadas por mulheres que passaram por tratamento de fertilidade para lidar com o processo de se tornarem mães e descrever seus relacionamentos com seus parceiros e profissionais de saúde durante todo esse processo.

**Métodos.:**

Este é um estudo qualitativo baseado na teoria fundamentada. Foram selecionadas 20 mulheres que passaram por Tecnologia de Reprodução Assistida (TRA) na Espanha. Entrevistas semiestruturadas foram utilizadas para coleta de dados. A análise seguiu métodos de teoria fundamentada.

**Resultados.:**

“Lutando por um sonho: a maternidade” é a principal categoria que descreve a luta das mulheres desde o diagnóstico de infertilidade até a gravidez e o parto bem-sucedidos. Esse processo permite que eles realizem o sonho de se tornarem pais de seus parceiros. Três subcategorias descrevem os diferentes estágios do processo: “Aceitando o tratamento: fazendo tudo o que você pode”, “Passando pelo tratamento: uma montanha-russa emocional” e “Repensando o sonho: desistindo ou reunindo forças para continuar?”

**Conclusão.:**

Mulheres submetidas à TRA enfrentam dificuldades ao tentar engravidar, pois não há garantias de que conseguirão realizar seu maior desejo na vida: a maternidade. As mulheres usam várias estratégias para melhorar o enfrentamento durante a TRA e continuam buscando apoio emocional em seu ambiente e/ou em mulheres que estão passando pela mesma situação.

## Introduction

Infertility is one of the main reproductive health problems in more economically developed countries, due in large part to the voluntary postponement of parenthood. The main causes of infertility in women are ovulation disorders, such as polycystic ovary syndrome, uterine disorders, and tubal disorders; in men, the most frequent causes of infertility are sperm disorders (morphology, concentration, mobility) and reproductive tract obstructions^1^ In recent years, these problems have become more frequent and, consequently, the demand for assisted reproductive technologies (ART) to treat fertility disorders has increased.^2^ Both infertility and ART have an impact on couples, but especially on women: before and during ART, women can display signs of deteriorating mental health and poor quality of life.^3^^,^^4^ Moreover, ART can lead to heightened levels of stress and anxiety among women,^4^ who tend to experience greater distress and fatigue than their male partners.^5^ Psychological adjustment appears to be related to women's cognitive representations of infertility and ART, such that the more negative they are, the more negative their emotional responses will be; and in addition, intense social pressure to have children causes them greater levels of distress.^6^ The need to have a child and the rejection of a childless lifestyle could become important predictors of anxiety and depression in the event that treatment is unsuccessful.^7^

Despite the harshness of the treatments, women often consider them a necessary sacrifice to have a child.^8^ However, when ART fails to deliver, women frequently report symptoms of depression and anxiety.^9^ Meanwhile, women who spend years receiving ART eventually accept the possibility that they will never have children, experiencing lower levels of stress and anxiety than women who have undergone a moderate number of treatments.^10^The experience of infertility and ART affects couples’ family and social lives and is influenced by sociocultural factors: race, ethnicity, religion, social class, etc. In pro-natalist countries, infertility is a greater problem as women are particularly valuable when they achieve the “status of mother”.^11^ Social and family relationships may also be affected due to the couple’s need to avoid painful situations such as children’s events, as well as the perceived lack of empathy with their situation from those around them.^12^

Several studies have sought to identify the risks associated with ART, as well as investigating ways to boost success rates in terms of pregnancies and live births.^13^ However, there has been little research into the experiences of women undergoing ART. Therefore, the aim of this study was to identify the strategies used by women who have undergone ART to cope with the difficulties they experienced and describe their relationships with their partners and the healthcare professionals who provided them with care throughout the process.

## Methods

This qualitative study takes a constructivist grounded theory approach.^14^ It is situated within the constructivist paradigm, which holds that reality is local and specifically constructed through action and considers people to be actors in the social world. Like classical grounded theory, this study adopts the theoretical perspective of symbolic interactionism. The study participants were 20 women aged 18-45 years old, who had received ART at public hospitals or specialist private clinics in Spain and had had a child in the last two years. No exclusion criteria were applied. In the first stage, purposive sampling was used and, as the study progressed, participants were selected based on their causes of infertility, maternal age, and years spent trying to conceive to complete the emerging categories. In the second stage, theoretical sampling was carried out to refine and develop the emerging categories. The recruitment of the participants was carried out by the first author face to face. The data were collected between September 2019 and February 2021 using semi-structured interviews that included the following items: Tell me about your experience with assisted reproduction treatment. Had you received other treatment previously or was it the first time? What type of treatment was it? Where did you carry it out? How did you feel?; Have you ever felt overwhelmed? What was it due to?; Have you felt the need to seek support from women who have gone through the same thing as you? In what way?; What was the relationship like with the healthcare personnel throughout the entire process?; How was your relationship with your partner been during the process? And with your family and friends? These questions offered the flexibility needed for participants to freely discuss their experiences. A pilot was carried out with four women. The interviews were conducted by the first author (credentials: woman, PhD, midwifery with training in the field), who had no previous relationship with the participants.

Due to the epidemiological situation resulting from the COVID-19 pandemic, 12 interviews were held via video call, while the remaining interviews were conducted at participants’ homes or cafés, depending on their preferences. No one else was present during the interview. The interviews lasted between 60 and 90 minutes. All interviews were recorded. Data collection ended when theoretical saturation was reached. No one refused to participate. No interview was repeated.

Field notes were made during and after the interviews. The interviews were transcribed in their entirety by the same researcher who conducted them to ensure the greatest possible precision. Data analysis was carried out concurrently alongside data collection using the constant comparative method. Firstly, open coding was performed using microanalysis, giving rise to *in vivo* codes such as ‘Doing everything possible’, ‘Ups and downs’, ‘Feeling supported’, and ‘Feeling incapable’. Subsequently, focused coding was conducted to give rise to categories and allow them to be combined and refined. Analytical and theoretical memos were written throughout the analysis to help guide the process and diagrams were produced to make it easier to identify relationships between categories, always seeking consistency with the data.. A single coder (the first author) carried out the coding of the data. The categories were not identified beforehand but were derived from the data. No software was used to manage data.

Various strategies were used to ensure the rigour of the study. Credibility was established by allowing the participants to freely express their experiences and by using their words to generate codes. Reliability was achieved through a detailed description of the selection process, taking the sociodemographic characteristics of the participants into consideration, and reporting the steps taken during the research process. A preliminary analysis of the first interviews was presented to the participants at a meeting; the information provided by them was used to review and confirm the emerging categories, enhancing the study’s relevance and credibility. Quotes from participants (identified by numbers) were presented to illustrate the categories. The first author maintained a reflexive stance, using a reflexive diary and field notes on theoretical and analytical aspects, which made the study more rigorous.

The research project was approved by the Ethics Committee of the Universidad Autónoma de Madrid (CEI-88-1644) and of the Hospital de Móstoles (CEIC 2018/014). All participants were informed, agreed to participate voluntarily, and signed the corresponding informed consent form. The confidentiality of the data and the anonymity of the participants were always guaranteed. Numeric codes were assigned to the verbatims extracted from each of the interviews. Only the first author Only the author had access to the personal data of the participants.

## Results

At the time of interview, most of the participants were aged 30-39 (70%) and took 2-4 years to conceive (50%). The most frequent cause of infertility was female (35%), The most frequent marital status was married (75%) and carried out the treatment in private hospitals (60%). Most of the participants completed university studies (40%) **(Table 1).**

**Table 1:** Characteristics of study of the 20 participants

Variable	Categories	*n*	%
Age	< 30 30-34 35-39 40 and more	2 7 7 4	10 35 35 20
Age of start seeking pregnant	< 30 30-34 35-39 40 and more	9 9 1 1	45 45 5 5
Highest level education completed	Primary school High school Vocational training University Education	2 4 6 8	10 20 30 40
Marital status	Married Cohabiting Single Divorced	15 3 1 1	75 15 5 5
Causes of infertility	Male infertility Female infertility Mixed infertility causes Unexplained infertility	6 7 5 2	30 35 25 10
Time to get pregnant	< 2 years 2-4 years > 4 years	4 10 6	20 50 30
Place of treatment	Public Hospital Private Hospital	8 12	40 60

**Figure 1:**
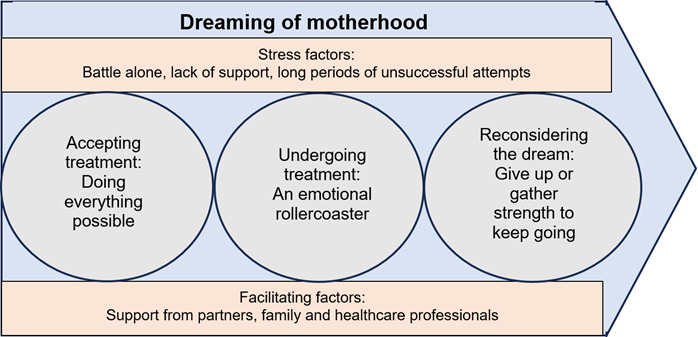
The process experienced by the women from their infertility diagnosis to their successful pregnancy

The process experienced by the women from their infertility diagnosis to their successful pregnancy, which constitutes **‘Dreaming of motherhood’** and enabled them and their partners to become parents, is described (Figure 1). Three subcategories point to the different stages in the process: ‘Accepting treatment: doing everything possible’, ‘Undergoing treatment: an emotional rollercoaster’, and ‘Reconsidering the dream: give up or gather strength to keep going?’. 

### Accepting treatment: doing everything possible

Once the women and their partners had acknowledged and accepted their infertility diagnosis, they decided to visit assisted reproduction clinics and accept the treatments offered to them. They made this decision after failing to conceive for long periods of time and experiencing an intense desire for parenthood, which they idealised over time, prompting the women to pursue the treatment(s) that would make it possible for them to fulfil their dream. They hoped and trusted that this treatment would be effective. The women accepted the treatment recommended to them by healthcare professionals, trusting that they would be offered the best option to achieve a long-awaited positive pregnancy test: *I was certain that I would be a mother, one way or another I would be a mother, for sure. I couldn’t imagine life with just the two of us... no, no way. It didn’t even cross my mind* (Informant 1; 33 years, 2 years to conceive, married). 

When a woman’s partner shared her desire to have children, this was a strong source of added motivation to embark on the proposed treatment and tackle the problem of infertility. The joint nature of this decision made the therapeutic process more tolerable: *He said to me: ‘You and I are going to be parents, no matter what, we’re going to be parents’. And he said: ‘I want you to get pregnant, I don’t want to adopt or... I want you to get pregnant and feel what it’s like to be pregnant’.* (Informant 6; 32 years, 7 months to conceive, married). When the couples had sufficient financial resources, they decided to commence treatment as quickly as possible. The intense desire expressed and demonstrated by the women drove them to seek to avoid the long waiting lists for treatment at public hospitals. In these situations, couples began ART at specialist private clinics with high pregnancy success rates in order to reduce the waiting time and fulfil their dream as soon as possible: *So we started the process, we didn’t think twice because I was so eager…* (Informant 17; 33 years, 3 years to conceive, married).

The study participants felt that they were the ones to suffer the physical and mental burden of treatment and that the burden was not shared with their partners, as most of the treatments involved medication for the women, who then suffered the resulting side effects. Treating infertility requires regular monitoring and visits to the clinics, which fall to the women to attend, sometimes alone: *It’s a huge burden... you do feel that the burden is on you, because you’re the one that has to get pregnant, you’re the one that has to take medication…* (Informant 2; 34 years; 2’5 years to conceive, cohabiting). Despite the women’s efforts and the extra burden that they carried, the desire to have children motivated them to continue their treatment, no matter how difficult and tiresome it could be. During treatment, the women were not concerned about the pain involved in certain techniques, such as egg retrieval, nor about the possible side effects of the medication prescribed; they were capable of prioritising their desire to conceive over their physical wellbeing: *I wanted to have a child and I didn’t care what I had to go through* (Informant 1). 

In order to conceive, the study participants not only adhered to the medication regimes that they were prescribed but also followed recommendations for a healthier lifestyle (balanced diet, moderate exercise, weight loss in the case of overweight or obesity, eliminating or reducing toxic habits such as smoking and alcohol, etc.) and endeavoured to rigorously comply with all the recommendations made by their healthcare professionals. The women also consulted other sources of information online and on social media, such as: different types of ART, steps to take, types of diagnostic tests that would provide more information, how to improve the results of ART, etc. When they searched for information online, this was largely because their healthcare professionals were not very approachable, their visits were short and mechanical, and they and their partners were given insufficient time to ask any questions they had. Knowledge and information about the treatments gave the women a sense of greater control over their situation and their destiny:*At the same time, I was researching elsewhere, and I have to say that if I hadn’t done that research and played an active role, I might not have got pregnant. I wouldn’t even have got pregnant. There’s a lot of ignorance around all this* (Informant 3; 28 years, 3 years to conceive, married).

The women were more active than their partners when it came to finding information about treatment and general care to achieve a successful pregnancy. When their partners did not cooperate with the recommendations for care and healthy habits or continued to engage in unhealthy habits such as smoking or failing to lose weight, the women were frustrated because they perceived these behaviours as an additional obstacle to pregnancy and to fulfilling their dream: *For example, in our case my partner smoked. We knew that smoking wouldn’t improve his sperm quality. You get angry every time they smoke a cigarette* (Informant 4; 37 years, 5 years to conceive, cohabiting). 

### Undergoing treatment: an emotional rollercoaster

Women undergoing treatment for infertility face an unfamiliar process, both in terms of the procedures carried out and the eventual outcome, which is not guaranteed to be successful. The process is described by the study participants as an emotional rollercoaster, characterised by mixed emotions and emotional ups and downs, with lows and highs experienced within the same day, and oscillation between hope and disappointment, uncertainty and fear, joy and sadness at different stages of treatment: *It’s not linear, like ‘Oh, I can’t do it’, you have emotional ups and downs, up, down, up, down (...). And they tell you it’s really... important for you to be optimistic and have a positive outlook. But of course, you’re positive, positive... and then it goes downhill. It’s really tough, I had a really hard time* (Informant 8; 35 years, 5 years to conceive, married).

At the start of their treatment, the women experienced hope and optimism, which was accentuated if they had received an infertility diagnosis with a positive prognosis or if they felt they were in the care of prestigious healthcare professionals with high pregnancy success rates. The availability of statistics on the probability of a successful pregnancy following ART had less of an influence than their hope in the prestigious professionals delivering their treatment. This may be why the couples experienced considerable disappointment after receiving a negative result at the start of their treatment: We were so hopeful, we were very naive, complete novices and we thought: ‘We’re at the best clinic, we’re undergoing the best treatment at * [private clinic], *we’re with the medical director of... I’m going to get pregnant; especially with PGD* [preimplantation genetic diagnosis] *and they’re telling me that my eggs are good quality and all that, I’m sure to get pregnant.’ We were so hopeful... and that was our mistake* (Informant 5; 39 years, 4 years to conceive, married). However, the women who had more information and were familiar with other women’s experiences of fertility treatment (friends or family members) were more aware of the success rates of different treatments. This knowledge of the experiences of women close to them made them hopeful but more realistic as they underwent the first treatment, as they were aware that the first procedure tends to fail. This cognitive coping strategy based on realistic thoughts and arguments made them more prepared for a negative result and normalising the situation helped them and their partners to cope with their disappointment and failure: *I mean, you start off well, the first one’s fine, you’re really hopeful. But when you do the test and it’s negative, you expect it because you understand that based on the percentages, they give you... it’s normal that you don’t get pregnant the first time* (Informant 2). 

During treatment, the women experienced intense emotions related to various factors. On the one hand, the lengthy waiting times between medical consultations raised the women’s stress levels as they felt that they were wasting time. On the other, good news (being perfectly placed to start treatment) often went hand-in-hand with bad news (failure of treatment despite optimal conditions). These mixed emotions were destabilising for the women, who experienced feelings of optimism and joy at the good news they received and disappointment and sadness when the treatment failed, all within the same treatment cycle: *Then comes the downer: ‘You’re perfect, your uterine lining is perfect, you’re a textbook case. I’ve got an incredible grade A blastocyst’. And then I don’t get pregnant again... so, it’s a situation I wouldn’t wish on anyone* (Informant 8). During the waiting time between embryo implantation or insemination and pregnancy testing, the women experienced uncertainty as they were unable to immediately ascertain the outcome of the treatment. Generally speaking, this uncertainty led to anxiety and desperation; the women experienced fear and concern that their actions might jeopardise the success of the treatment and sadness at the thought that they might receive a negative result: *During those 12 days, I think it was, before they did the test, I did a load of tests myself…* (Informant 1). 

The participants who successfully conceived after the first or second attempt considered themselves to be lucky, as one woman said, as they had not had to undergo lengthy treatment processes like other women, which constituted sufficient reason to complain about the process: *We were very lucky that on the first attempt... there you have it.* (Informant 11; 34 years, 3 years to conceive, married). However, the couples who had undergone several successive treatments and obtained negative results each time felt frustration, sadness, and sometimes guilt. Their frustration was triggered by being unable to understand why the treatments they had undergone had failed. The women sometimes experienced guilt if unforeseen events during the treatment cycle had caused them added stress or if they thought they had engaged in an activity that could have had a harmful effect. In response to their failure to conceive, they sought personal causes and this heightened their feelings of guilt and frustration. The negative results of the treatments distanced the women still further from their goal of motherhood: *And then, [there’s] even a sense of guilt. Because you often start to think: ‘Why hasn’t it stuck? Is it because I had chips?’* (Informant 4).

The failure to achieve motherhood reinforced a more negative experience and the idea that the women’s bodies were unfit as they were unable to achieve or maintain pregnancy. The need to find an explanation prompted them to consider the possibility that there was a problem that had not yet been diagnosed or identified, causing them to distrust the treatments and healthcare professionals. Attributing failure to the treatments or professionals delivering them was a strategy that allowed the women to remain hopeful that they would be able to conceive: *The last three or four months were like: ‘No, we’re not going to achieve it, it’s not working, something’s wrong’. My thoughts were: ‘Something’s wrong, something’s up that you can’t see and that... that’s making it not work’* (Informant 2). 

During treatment, if the couple were treated in a cold, dehumanising manner by healthcare professionals or if care was solely focused on the woman, they had a more negative experience; they felt misunderstood and frustrated, as well as considering that their emotional needs throughout the process had been overlooked. On the contrary, when they were given an opportunity to experience the entire treatment process with their partners, the women had a more positive experience as it made the process feel more natural and more like a spontaneous pregnancy: *In my case, they didn’t even look at my husband at the visits. He’d be asking questions and they’d look at him and reply like: ‘You have no right to ask questions’. It was all centred around the mother, which is great but he’s just as important as me, the process is about both of us and he’s 50% of it* (Informant 11).

Support from partners was a key factor influencing the women’s experiences. The couples who approached treatment as a shared problem and struggle described how the situation had strengthened the bond between them. However, when the members of the couple held different attitudes to treatment and the woman felt like she was fighting a battle alone, the couples were plunged into crisis. A lack of understanding and communication within the couple made it harder to express their feelings and give and receive support. *Sometimes he’d say: ‘you’re obsessed with it’. I’d say to him: ‘No, if you had it your way, we might still be at the first clinic’* (Informant 3). 

The couples’ families and friends had an impact on their experiences of ART. Support from family and friends helped make the process less stressful. However, despite having a strong support network, not all the couples wanted to share the news that they were undergoing treatment and avoided discussing it with the people close to them to reduce stress. In some cases, they even pretended that they were fine despite their treatment failing to save family members and even their partners from worrying about them. Looking for support from women who had gone through the same experience and reading their stories helped them cope with their failed treatment: *It depends on each person’s experience, the people around you, the support you have... financial support, emotional support, how you want to tell people about it. Because you spend the whole day talking about it, it’s your whole life, and that’s difficult. If you don’t talk about it it’s like you’re withdrawing into yourself, if you talk about it it’s like... people don’t know how to talk about it, they don’t know what to say* (Informant 18, 42 years, 2 years to conceive, divorced).

### Reconsidering the dream: give up or gather strength to keep going?

Women who experienced multiple failures to conceive weighed up different options and employed a variety of strategies. Some considered abandoning treatment as they were overwhelmed and found themselves unable to repeat the same cycles and relive the same experiences. The physical and mental burden on women during ART is intense. Some participants reached the conclusion that it was not worth the suffering and questioned whether to continue with the treatment or give up as they felt unable to cope - both physically and psychologically - with another treatment and another possible failure. The study participants considered different options: giving up and abandoning treatment or having a break before continuing their struggle. The idea of giving up was stronger in older women and women who had undergone more treatments. 

The women opted to have a break to recover before continuing to fight to conceive; they thought that this would allow them to control their emotions, gather strength, and prevent the quest for pregnancy from taking over their entire lives: *I’d set a deadline, a time limit. I was very clear on that: if that last time didn’t work, I wouldn’t be a mother and that would be fine, that would be it* (Informant 18). Breaks between one treatment and another is a strategy that the women believed would enhance the efficacy of the treatment. In their thoughts and reflections, they wondered whether their bodies were not responding adequately to the treatment due to their physical and mental exhaustion, making a break from treatment necessary: *My body was completely exhausted by that point. I’d had almost 90 injections; the third stimulation took a long time because the follicles were growing much slower. I was exhausted from travelling so much, from so many injections. I was a bit... I was carrying a lot of baggage, and my body wasn’t responding as it should* (Informant 6). 

## Discussion

The results of this study reveal the difficult process experienced by women undergoing ART as they attempt to achieve a successful pregnancy, as they must live with the uncertainty and fear that they will be unable to fulfil their greatest wish: motherhood. In this situation, ART becomes a priority that takes precedence over other areas of their lives. Women who are eager to conceive after long periods of unsuccessful attempts opt for treatment at private clinics to start as soon as possible. This is likely to be associated with the limited resources for ART in the public health system.^15^ This study shows that women seek information online to acquire the skills they need to handle ART, understand the steps involved, and learn how to boost their chances of a positive result, reinforcing the idea that women continue to feel responsible for reproduction and tackling infertility. This finding is corroborated by other studies. Weissman *et al*. ^16^ showed that women look for more information online than men, regardless of the cause of infertility. Mayette *et al*.^17^ also point out that although women preferred to use the information provided by the health professionals who cared for them, this was felt to be insufficient and they would turn to other sources to better understand the whole process they were going through, especially with regard to mental health support during treatment. ART places a considerable physical and mental burden on women. This is apparent both in women who are accepting that they need treatment and those who are undergoing treatment. In some cases, they need to take breaks as a strategy to increase the efficacy of treatment or they consider abandoning treatment altogether if they do not become pregnant on the next attempt. The results of this study indicate that one of the key factors prompting women to abandon treatment may be a loss of hope of ever having a successful pregnancy. Stress experienced by women undergoing treatment and financial difficulties are reasons for treatment drop-out reported in the literature.^18^^,^^19^ Research has also shown that some women, despite experiencing an intense desire to abandon or pause their treatment, feel forced to continue due to the pressure placed upon them by healthcare professionals at clinics.^20^

This study shows that women who do not consider abandoning treatment feel a duty to do everything possible to have a child, as they do not want to give up on this plan for their lives. The existence of more effective treatments, such as egg donation or gamete donation, encourage women to continue the process by keeping the hope that they will eventually have children alive as they have not yet exhausted all treatment options. Copp *et al*.^21^ demonstrate that the decision on whether to continue treatment is related, among other factors, to perceived social pressure and fear of future regret at not having fought to have children. Doing everything possible to access motherhood reflects women’s need to adhere to established social norms and fit into the social construct of “woman=mother”.^22^In the work of Fusco et al, participants described their bodies as the repository of their hopes, but also as impaired, defective and non-functional, thus forming a ‘paradoxical’ representation. This links to the socially derived meanings of infertility since the expectations women expressed were that their bodies were born to procreate.^23^ Couples undergoing ART should be given information about the process in accessible language and sufficient time at their medical consultations to answer all their questions, as this study has highlighted. They also appreciate being given “bad news” in an appropriate manner and receiving emotional support from healthcare professionals who are pleasant and show empathy. These results corroborate those obtained by other researchers.^24^^,^^25^

This study also found that a lack of personalised care led to greater emotional distress among couples and resulted in distrust of the healthcare professionals caring for them. Meanwhile, some men felt overlooked and even ignored at medical consultations when care was exclusively centred on women. As in other studies, these results show that sharing hopes, disappointments and pain with partners can help to strengthen couple bonds and influence women's experiences, making it easier for them to cope.^26^In addition, couples feel it is very important to work together to build the parent-child relationship and achieve the goal of having a child.^25^

Regarding the limitations of the study, given the importance of personal interaction and contact for data collection in qualitative research, the data may have been influenced by the virtual format of the interviews during the COVID-19 pandemic. Above all, however, the health and wellbeing of the participants and researchers was prioritised, and the personal situation of the participants was checked before each interview to confirm that they were still willing to participate.^27^ Meanwhile, it is possible that the results may differ in other sociocultural contexts where ART is more accessible in the public health system or where there is less social pressure to have children. 

Future research should explore the experiences of women with secondary infertility, as their experiences may be different having previously had children naturally. It is also relevant to explore couples’ experiences of ART, including same-sex couples, as well as those of women who decide to undergo treatment without a partner.

Conclusion. Motherhood is a dream that the women in this study wished to fulfil. As a result, they decided to undergo ART to achieve this goal after accepting their infertility diagnosis. Women use various strategies to improve coping during ART: they seek information to help them understand and manage the problem and improve treatment outcomes, and they continue to seek emotional support from their peers and/or women who are experiencing the same situation. Similarly, feeling that it is a shared battle with their partner and having empathetic health professionals also facilitates coping during the process. They also use different tactics to adapt to motherhood, such as dedicating time to self-care or going to health professionals for support and emotional support. Co-responsibility for the care of the child also helps women to adapt, as they do not feel alone in caring for the new member of the family. In addition, the ordeal of treatment can help women to develop resilience, helping them to cope better with all the hard times of motherhood. Health professionals must attend to the emotional needs of women in a systematic and protocolised way, so that their quality of life is not affected. Midwives and obstetricians, because of their closeness to women during pregnancy, childbirth and postpartum, have a key role in attending to women's needs. They should value maternal wellbeing, promote the expression of feelings and facilitate strategies that allow them to face this process in the best possible way, as well as discourage actions that may encourage stress and anxiety in women.

## References

[1] World Health Organization [WHO] (2024). Infertility.

[2] Sociedad Española de Ginecología y Obstetricia [SEGO] Técnicas de Reproducción Asistida.

[3] Chachamovich JR, Chachamovich E, Ezer H, Fleck MP, Knauth D, Passos EP (2010). Investigating quality of life and health-related quality of life in infertility: a systematic review. Journal of Psychosomatic Obstetrics & Gynecology.

[4] Kato T, Sampei M, Saito K, Morisaki N, Urayama KY (2021). Depressive symptoms, anxiety, and quality of life of Japanese women at initiation of ART treatment. Scientific Reports.

[5] Omani R, Maroufizadeh S, Ghaheri A, David B (2018). Generalized Anxiety Disorder-7 (GAD-7) in people with infertility: A reliability and validity study. Middle East Fertility Society Journal.

[6] Moutzouri M, Sarantaki A, Gourounti K (2021). The association of cognitive representations with psychological adjustment in experience of infertility and fertility treatment: A systematic review. European Journal of Midwifery.

[7] Gourounti K, Anagnostopoulos F, Vaslamatzis G (2011). Psychometric properties and factor structure of the Fertility Problem Inventory in a sample of infertile women undergoing fertility treatment. Midwifery.

[8] Langher V, Fedele F, Caputo A, Marchini F, Aragona C (2019). Extreme Desire for Motherhood: Analysis of Narratives from Women Undergoing Assisted Reproductive Technology (ART). European Journal of Psychology.

[9] Maroufizadeh S, Karimi E, Vesali S, Omani Samani R (2015). Anxiety and depression after failure of assisted reproductive treatment among patients experiencing infertility. International Journal of Gynecology & Obstetrics.

[10] Boivin J, Takefman J, Tulandi T, Brender W (1995). Reactions to infertility based on extent of treatment failure. Fertility and Sterility.

[11] Greil AL, Slauson-Blevins K, McQuillan J (2010). The experience of infertility: a review of recent literature. Sociology of Health & Illness.

[12] López-Criado S (2017). Reproducción asistida: profesionales que ayudan a cumplir un sueño. Metas de Enfermería.

[13] Gullo G, Scaglione M, Laganà AS, Perino A, Andrisani A, Chiantera V (2023). Assisted Reproductive Techniques and Risk of Congenital Heart Diseases in Children: a Systematic Review and Meta-analysis. Reproductive Sciences.

[14] Charmaz K (2014). Constructing Grounded Theory.

[15] Núñez-Calonge R (2017). Problemas éticos en reproducción asistida. Revista Iberoamericana de Fertilidad y Reproducción Humana.

[16] Weissman A, Gotlieb L, Ward S, Greenblatt E, Casper RF (2000). Use of the internet by infertile couples. Fertility and Sterility.

[17] Mayette E, Scalise A, Li A, McGeorge N, James K, Mahalingaiah S (2024). Assisted reproductive technology (ART) patient information-seeking behavior: a qualitative study. BMC Womens Health.

[18] Domar AD, Rooney K, Hacker MR, Sakkas D, Dodge LE (2018). Burden of care is the primary reason why insured women terminate in vitro fertilization treatment. Fertility and Sterility.

[19] Ebrahimzadeh Zagami S, Latifnejad Roudsari R, Janghorban R, Allan HT (2022). Trying for a second chance: Iranian infertile couples' experiences after failed ART. Journal of Psychosomatic Obstetrics & Gynecology.

[20] Carson A, Webster F, Polzer J, Bamford S (2021). The power of potential: Assisted reproduction and the counter stories of women who discontinue fertility treatment. Social Science and Medicine.

[21] Copp T, Kvesic D, Lieberman D, Bateson D, McCaffery KJ (2020). 'Your hopes can run away with your realistic expectations': a qualitative study of women and men's decision-making when undergoing multiple cycles of IVF. Human Reproduction Open.

[22] Oliva-Bello K, Batista J (2019). Infertilidad femenina y modos de subjetivación: cuando el yo se percibe fallido. Integración Integración Academica en Psicología.

[23] Fusco C, Masaro C, Calvo V (2024). 'I feel like I was born for something that my body can't do': a qualitative study on women's bodies within medicalized infertility in Italy. Psychol Health.

[24] Kim M, Yi SJ, Hong JE (2020). Experiences of Women with Male Factor Infertility under In Vitro Fertilization. International Journal of Environmental Research and Public Health.

[25] Tseng YM, Mu PF, Lai YM (2024). Hope experiences in pregnant women after artificial reproduction technology: Becoming a mother. Nurs Health Sci.

[26] Boz İ, Teskereci G, Akgün M (2021). The experience of becoming a mother following successful in vitro fertilization: A grounded theory. Journal of Advanced Nurse.

[27] Hernán-García M, Lineros-González C, Ruiz-Azarola A (2020). Cómo adaptar una investigación cualitativa a contextos de confinamiento. Gaceta Sanitaria.

